# Assessment of Depression and Its Association With Sleep Quality Among the General Population of Perambalur in Tamil Nadu, India: A Cross-Sectional Analysis

**DOI:** 10.7759/cureus.79853

**Published:** 2025-02-28

**Authors:** Shivasakthimani R, Tamilarasan Muniyapillai, Aswin A, Mugil Sriram, Mugunthan K, Naveen N, Naveen SG, Naveen Kumar

**Affiliations:** 1 Community Medicine, Dhanalakshmi Srinivasan Medical College and Hospital, Siruvachur, IND

**Keywords:** cross-sectional studies, depression, epidemiology, mental health, sleep quality, sleep wake disorders

## Abstract

Background

Sleep quality and depression represent significant public health concerns with complex bidirectional relationships. Despite extensive research in specific populations, comprehensive studies examining their association with general populations remain limited, particularly in developing regions. This study aimed to assess the prevalence of depression and poor sleep quality among the general population of Perambalur, Tamil Nadu, examine their interrelationship, and identify associated sociodemographic and behavioral factors influencing this relationship. Additionally, the study sought to analyze the impact of depression severity on sleep quality parameters and investigate potential risk factors affecting both conditions.

Methodology

A cross-sectional analytical study was conducted among 650 participants from Perambalur district, Tamil Nadu, India. Data collection involved face-to-face interviews using a structured questionnaire comprising sociodemographic profiles, the Pittsburgh Sleep Quality Index (PSQI) for sleep quality assessment, and the Patient Health Questionnaire-9 (PHQ-9) for depression evaluation. The questionnaire underwent forward-backward translation and pilot testing. Sleep quality was categorized using PSQI scores (>5 indicating poor sleep), while depression severity was classified as minimal (0-4), mild (5-9), moderate (10-14), moderately severe (15-19), and severe (20-27). Data analysis employed descriptive statistics, chi-square tests, and multivariable logistic regression, with p<0.05 considered statistically significant.

Results

The study population, with a mean age of 35.71±14.70 years, comprised 381 (58.6%) females and 269 (41.4%) males. The depression analysis revealed that 385 (59.2%) participants had minimal depression, 171 (26.3%) had mild depression, 62 (9.5%) had moderate depression, 27 (4.2%) had moderately severe depression, and five (0.8%) had severe depression. Poor sleep quality was reported by 142 (21.8%) participants. Sleep-related parameters showed 100 (15.4%) participants experiencing difficulty initiating sleep, 102 (15.7%) reporting midnight awakenings, and 34 (5.2%) using self-medication. Logistic regression identified self-medication (adjusted odds ratio (AOR)=8.45, 95% CI: 3.12-22.86) and moderately severe/severe depression (AOR=7.92, 95% CI: 3.45-18.19) as the strongest predictors of poor sleep quality. PSQI scores demonstrated progressive deterioration across depression severity levels, increasing from 3.2±1.8 in minimal to 11.3±3.2 in severe depression. A strong positive correlation was observed between PSQI and PHQ-9 scores (r=0.65, p<0.001).

Conclusion

The study establishes significant associations between depression severity and sleep quality, highlighting the need for integrated healthcare approaches. The identified sociodemographic risk factors and high prevalence of self-medication underscore the importance of targeted interventions and improved access to professional healthcare services, particularly focusing on vulnerable populations in both urban and rural settings.

## Introduction

The Global Burden of Disease study revealed a substantial escalation in depression-related morbidity from 172 million cases in 1990 to 258 million cases in 2017, empirically demonstrating the increasing prevalence and global health impact of this condition while underscoring its significance as an interrelated public health concern with sleep quality [1]. The bidirectional relationship between these conditions manifests through various mechanisms: sleep disturbances, characterized by difficulties in sleep initiation, maintenance, early morning awakenings, and diminished quality, frequently precede depressive symptoms while simultaneously serving as common manifestations of depressive disorders [2]. These disruptions exert deleterious effects on cognitive functioning, particularly affecting attention, working memory, and executive functions [3-5], which subsequently compromises productivity, increases accident risk, and diminishes quality of life. Moreover, the neurobiological and psychological pathways connecting sleep dysregulation and depression involve complex interactions between stress mechanisms, arousal systems, and coping strategies [6], with evidence suggesting that persistent insomnia may function as a perpetuating factor for depression in specific demographic contexts [7].

Despite the growing corpus of international evidence describing sleep-depression associations across diverse populations, ranging from Brazilian cross-sectional analyses revealing significant sleep quality issues [8] to Indonesian studies demonstrating a considerable prevalence of both conditions [9], a critical knowledge gap persists regarding the contextual manifestation of these relationships within geographically and culturally distinct settings. The impact of these conditions transcends individual health outcomes to affect broader societal functioning through decreased academic performance [10,11], increased occupational stress [12], and compromised quality of life [13]. Additionally, chronic sleep disruptions correlate with elevated risks for physical health complications including hypertension, metabolic disorders [14,15], and T2DM, further amplifying their public health significance. This multifaceted impact underscores the necessity for a comprehensive examination of these conditions, particularly in underrepresented populations.

The present investigation addresses this research gap by examining the specific relationship between depression and sleep quality among the general population of Perambalur, India, while simultaneously assessing various sociodemographic and contextual factors that potentially moderate this association. Through rigorous methodological approaches utilizing validated screening instruments, this study aims to elucidate population-specific manifestations of the sleep-depression relationship while generating actionable insights for healthcare providers and policymakers. The findings will contribute substantively to the existing knowledge base by providing culturally contextualized evidence regarding the interface between these conditions, potentially informing the development of targeted interventions simultaneously addressing both sleep disturbances and depression within this unique population context. Such evidence is essential for developing integrated approaches to mental health that acknowledge the interdependence of sleep quality and psychological well-being.

## Materials and methods

Study design and study setting

A cross-sectional analytical study was conducted among the general population residing in Perambalur district, Tamil Nadu, India.

Study period

The study was conducted over a period of three months from December 2023 to February 2024.

Ethics committee approval

The study protocol was approved by the Institutional Ethics Committee of Dhanalakshmi Srinivasan Medical College, Perambalur (IECHS/IRCHS/No. 316). Written informed consent was obtained from all participants after explaining the study objectives and procedures. Confidentiality and anonymity of the participants were maintained throughout the study.

Inclusion criteria

Adults aged 18 years and above, permanent residents of the Perambalur district for at least six months, and those willing to participate in the study were included. Both male and female participants who could comprehend and respond to the questionnaire were eligible for participation.

Exclusion criteria

Individuals with diagnosed psychiatric disorders (except depression), those on medications affecting sleep patterns, pregnant women, shift workers, and those unable to provide informed consent were excluded from the study. Participants with incomplete questionnaire responses were also excluded from the final analysis.

Sample size estimation

The sample size was calculated using Cochran's formula: n = Z² × p × (1-p) / d², where Z is the standard normal variate (1.96 at 95% confidence level), p is the expected prevalence, and d is the absolute precision. Based on the study by Alfian et al. (2024) conducted among the general Indonesian population [9], which reported a depression prevalence of 35%, and considering an absolute precision of 4%, the minimum sample size was calculated as 546. Accounting for a 20% non-response rate, the final sample size was determined to be 650 participants.

Sampling method

The study utilized a convenience sampling technique for participant recruitment, acknowledging both methodological considerations and practical constraints of community-based research. The sampling process was executed in two distinct phases. Initially, the district was stratified into urban and rural areas to ensure representation from both settings, and accessible areas within each stratum were identified based on logistical feasibility and population density. Subsequently, eligible participants were recruited through consecutive sampling from the identified areas based on their availability and willingness to participate. To minimize selection bias, recruitment was conducted at varying times of the day and different days of the week, continuing until the calculated sample size was achieved.

Data collection procedure

Data collection was conducted by trained research assistants through face-to-face interviews using a structured questionnaire. The questionnaire was initially developed in English and translated to the local language following standard forward-backward translation procedures. A pilot study was conducted among 30 participants (not included in the final analysis) to assess the feasibility and reliability of the questionnaire. Based on the pilot study feedback, necessary modifications were made to improve clarity and comprehension.

The questionnaire comprised three sections: sociodemographic profile, sleep quality assessment, and depression assessment. The sociodemographic section included variables such as age, gender, education level (categorized as higher education, school education, and no formal education), occupation (organized work, unorganized work, and unemployed), region (rural/urban), and family size. Sleep-related variables included time to fall asleep, midnight awakening, occurrence of bad dreams, use of sleep medication, and level of enthusiasm.

Study tools

Sleep quality was assessed using the Pittsburgh Sleep Quality Index (PSQI), a validated tool widely used in sleep research [16-18]. The PSQI evaluates seven components of sleep quality over the past month: subjective sleep quality, sleep latency, sleep duration, habitual sleep efficiency, sleep disturbances, use of sleep medication, and daytime dysfunction. The global PSQI score ranges from 0 to 21, with scores >5 indicating poor sleep quality [16].

Depression was evaluated using the Patient Health Questionnaire-9 (PHQ-9), a reliable screening tool for detecting depression severity [2,18]. The PHQ-9 consists of nine items scored on a four-point Likert scale (0-3), with total scores ranging from 0 to 27. Depression severity was categorized as minimal (0-4), mild (5-9), moderate (10-14), moderately severe (15-19), and severe (20-27). PHQ-9 was selected as the primary depression assessment instrument due to its superior psychometric properties, demonstrated cross-cultural validity, and pragmatic implementation advantages. Furthermore, the PHQ-9 has been extensively validated in Indian populations, demonstrating robust cultural applicability with high internal consistency (Cronbach's α = 0.89) and test-retest reliability (r = 0.84). Its free availability without licensing requirements and established translation protocols into Tamil enhanced its methodological appropriateness for this community-based study in Perambalur.

Data analysis

Data analysis was performed using SPSS version 26.0 (IBM Corp., Armonk, NY). Descriptive statistics were presented as frequencies and percentages for categorical variables, while continuous variables were expressed as mean ± SD. The chi-square test was used to assess the association between categorical variables, with p<0.05 considered statistically significant. The relationship between depression and sleep quality was analyzed using multivariable logistic regression. The dependent variable was dichotomized as good sleep quality (PSQI ≤5) and poor sleep quality (PSQI >5). Independent variables included depression severity, sociodemographic characteristics, and sleep-related parameters. Crude and adjusted odds ratios (AOR) with 95% CI were calculated to quantify the strength of associations. To control for potential confounding factors, variables showing significant association in bivariate analysis (p<0.05) were included in the multivariate model. The model's goodness of fit was assessed using the Hosmer-Lemeshow test. Multicollinearity among independent variables was evaluated using variance inflation factors (VIF).

## Results

Table 1 shows the sociodemographic characteristics, depression severity, and sleep quality parameters. The study population comprised 650 participants with a mean age of 35.71 years (SD=14.70). The sample demonstrated a slight female predominance, with 381 (58.6%) female participants compared to 269 (41.4%) male participants. Regarding educational status, a majority of participants had completed higher education (480 (73.8%)), while 111 (17.1%) had school education, and 59 (9.1%) reported no formal education. Occupational distribution revealed that 222 (34.2%) participants were engaged in organized work sectors, 181 (27.8%) in unorganized work, and 247 (38.0%) were unemployed. The study population was predominantly urban-based (381 (58.6%)) compared to rural residents (269 (41.4%)). Family size analysis showed that most participants (429 (66.0%)) belonged to households with 4-5 members, followed by families with ≤3 members (181 (27.8%)), and a smaller proportion with >5 members (40 (6.2%)). Depression severity assessment revealed that minimal depression was most prevalent (385 (59.2%)), followed by mild depression (171 (26.3%)). More severe categories showed decreasing frequencies: moderate depression (62 (9.5%)), moderately severe depression (27 (4.2%)), and severe depression (5 (0.8%)). Sleep quality evaluation using the PSQI indicated that while the majority reported good sleep quality (508 (78.2%)), a considerable proportion experienced poor sleep (142 (21.8%)). Specific sleep-related parameters revealed that 100 (15.4%) participants reported difficulty initiating sleep within 30 minutes, and 102 (15.7%) experienced midnight awakenings. Additionally, 68 (10.5%) participants reported sleep disruption due to bad dreams, while 34 (5.2%) admitted to using self-medication for sleep. Notably, the majority of participants (583 (89.7%)) reported maintaining enthusiasm, with only 67 (10.3%) indicating a lack thereof.

**Table 1:** Sociodemographic characteristics, depression severity, and sleep quality parameters among the general population of Perambalur, Tamil Nadu (n=650) *Mean ± SD. PSQI, Pittsburgh Sleep Quality Index; PHQ-9, Patient Health Questionnaire-9

Variables		Frequency	Percentage
Age in years (mean ± SD)		35.71±14.70*	
Gender	Male	269	41.4
	Female	381	58.6
Education	Higher education	480	73.8
	School education	111	17.1
	No formal education	59	9.1
Occupation	Organized work	222	34.2
	Unorganized work	181	27.8
	Unemployed	247	38.0
Region	Rural	269	41.4
	Urban	381	58.6
Number of family members	≤3	181	27.8
	4-5	429	66.0
	>5	40	6.2
PHQ-9	Minimal depression	385	59.2
	Mild depression	171	26.3
	Moderate depression	62	9.5
	Moderately severe depression	27	4.2
	Severe depression	5	0.8
PSQI	Good sleep	508	78.2
	Poor sleep	142	21.8
Not able to sleep within 30 minutes	Yes	100	15.4
	No	550	84.6
Wake in the middle of the night	Yes	102	15.7
	No	548	84.3
Wake up due to bad dreams	Yes	68	10.5
	No	582	89.5
Using self-medication for sleep	Yes	34	5.2
	No	616	94.8
Enthusiasm	Yes	583	89.7
	No	67	10.3

Table 2 shows the comprehensive analysis that examined the relationship between depression status and various sociodemographic and sleep-related parameters among the study population. The mean age differed significantly between groups, with non-depressed individuals being older (38.38±13.99 years) compared to those with depression (31.86±14.88 years) (p<0.001). Gender distribution analysis showed that among males, 235 (61.7%) had no depression while 146 (38.3%) had depression. Among females, 150 (55.8%) had no depression while 119 (44.2%) had depression, though this difference was not statistically significant (p=0.130). The educational status analysis demonstrated varying depression rates across educational levels, with higher education participants showing 279 (57.5%) without depression and 204 (42.5%) with depression. The school education category showed 76 (68.5%) without depression and 35 (31.5%) with depression, while those with no formal education had 33 (55.9%) without depression and 26 (44.1%) with depression (p=0.091).

**Table 2:** Association between sociodemographic characteristics, sleep parameters, and depression status among adults in Perambalur, Tamil Nadu: a comparative analysis (n=650) *Represented in mean ± SD, and an independent T-test was used. ^#^The chi-square test was used. Statistically significant when the p-value was less than 0.05.

Variables		No Depression	Depression	Chi-square value	p-value
Age in years*		38.38 ± 13.99	31.86 ± 14.88		<0.001
Gender	Male	235 (61.7)	146 (38.3)	2.287	0.130
	Female	150 (55.8)	119 (44.2)		
Education	Higher Education	279 (57.5)	204 (42.5)	4.784	0.091
	School Education	76 (68.5)	35 (31.5)		
	No formal Education	33 (55.9)	26 (44.1)		
Occupation	Organized Work	149 (67.1)	73 (32.9)	55.044	<0.001^#^
	Unorganized Work	134 (74.0)	47 (26.0)		
	Unemployed	102 (59.2)	145 (58.7)		
Region	Rural	179 (65.4)	93 (34.6)	7.298	0.007^#^
	Urban	209 (54.9)	172 (45.1)		
Number of family members	≤ 3	99 (54.7)	82 (45.3)	2.154	0.341
	4-5	262 (61.1)	167 (38.9)		
	>5	24 (60)	16 (40)		
Not able to sleep within 30 minutes	Yes	20 (20)	80 (80)	75.322	<0.001^#^
	No	365 (66.4)	185 (33.6)		
Wake in the Middle of the Night	Yes	22 (21.6)	80 (78.4)	71.066	<0.001^#^
	No	363 (66.2)	185 (33.8)		
Wake up due to bad dreams	Yes	9 (13.2)	59 (86.8)	66.535	<0.001^#^
	No	376 (64.6)	206 (35.4)		
Using self-medication for sleep	Yes	5 (14.7)	29 (85.3)	29.454	<0.001^#^
	No	380 (61.7)	236 (38.3)		
Enthusiasm	Yes	376 (64.5)	207 (35.5)	64.883	<0.001^#^
	No	9 (13.4)	58 (86.6)		

Occupational status showed highly significant associations (p<0.001), with organized work showing 149 (67.1%) without depression versus 73 (32.9%) with depression. Unorganized work demonstrated 134 (74.0%) without depression versus 47 (26.0%) with depression, while unemployment showed notably different patterns with 102 (59.2%) without depression versus 145 (58.7%) with depression. Sleep-related parameters demonstrated strong associations with depression status. Difficulty in falling asleep within 30 minutes was significantly associated with depression (p<0.001), with 80 (80%) of those reporting this difficulty having depression. Similarly, middle-of-night awakening showed a significant association (p<0.001), with 80 (78.4%) of those experiencing it having depression. Bad dreams were also significantly associated with depression (p<0.001), with 59 (86.8%) of those reporting bad dreams having depression.

Self-medication for sleep showed a significant association with depression status (p<0.001), where 29 (85.3%) of those using sleep medication had depression. Notably, enthusiasm levels were significantly associated with depression (p<0.001), with 58 (86.6%) of those reporting a lack of enthusiasm having depression, compared to only 207 (35.5%) among those with enthusiasm. Regional analysis revealed significant differences (p=0.007), with urban residents showing higher depression rates (172 (45.1%)) compared to rural residents (93 (34.6%)). Family size did not show a significant association with depression status (p=0.341), with relatively similar distribution across different family size categories.

Table 3 shows the association between sociodemographic characteristics, sleep-related parameters, and sleep quality. The mean age significantly differed between good and poor sleep quality groups (36.74±14.25 years vs. 32.03±15.73 years, respectively; p<0.001), suggesting age-dependent variations in sleep patterns.

**Table 3:** Association between sociodemographic characteristics, sleep-related parameters, and sleep quality among the general population of Perambalur district (N=650) *Represented in mean ± SD, an independent T-test was used, and statistically significant when the p-value was less than 0.05. ^#^The chi-square test was used and statistically significant when the p-value was less than 0.05.

Demographic		Good sleep	Poor sleep	Chi-square value	p-value
Age in years*		36.74±14.25	32.03±15.73		<0.001
Gender	Male	301 (79.0)	80 (21.0)	0.388	0.533
	Female	207 (77.0)	62 (23.0)		
Education	Higher education	374 (77.9)	106 (22.1)	0.099	0.952
	School education	88 (79.3)	23 (20.7)		
	No formal education	46 (78.0)	13 (22.0)		
Occupation	Organized work	189 (85.1)	33 (14.9)	27.985	<0.001^#^
	Unorganized work	153 (84.5)	28 (15.5)		
	Unemployed	166 (67.2)	81 (32.8)		
Region	Rural	214 (79.6)	55 (20.4)	0.527	0.468
	Urban	294 (77.2)	87 (22.8)		
Number of family members	≤3	136 (75.1)	45 (24.9)	8.580	0.014^#^
	4-5	347 (80.9)	82 (19.1)		
	>5	25 (62.5)	15 (37.5)		
Not able to sleep within 30 minutes	Yes	30 (30)	70 (70)	160.505	<0.001^#^
	No	478 (86.9)	72 (13.1)		
Wake in the middle of the night	Yes	40 (39.2)	62 (60.8)	107.438	<0.001^#^
	No	468 (85.4)	80 (14.6)		
Wake up due to bad dreams	Yes	14 (20.6)	54 (79.4)	147.401	<0.001^#^
	No	494 (84.9)	88 (15.1)		
Using self-medication for sleep	Yes	1 (2.9)	33 (97.1)	118.869	<0.001^#^
	No	507 (82.3)	109 (17.7)		
Enthusiasm	Yes	494 (84.7)	89 (15.3)	143.440	<0.001^#^
	No	14 (20.9)	53 (79.1)		

Occupational status demonstrated a highly significant association with sleep quality (χ²=27.985, p<0.001). Participants engaged in organized and unorganized work sectors exhibited better sleep quality profiles (85.1% and 84.5% reporting good sleep, respectively) compared to unemployed individuals, among whom only 166 (67.2%) reported good sleep quality. Household composition significantly influenced sleep quality (χ²=8.580, p=0.014), with optimal sleep patterns observed in households of 4-5 members (347 (80.9%) reporting good sleep).

Sleep-related behavioral parameters showed robust statistical associations with overall sleep quality. Among participants reporting difficulty initiating sleep within 30 minutes, 70 (70.0%) experienced poor sleep quality compared to only 72 (13.1%) among those without this difficulty (χ²=160.505, p<0.001). Similarly, nocturnal awakening patterns significantly correlated with sleep quality (χ²=107.438, p<0.001), with 62 (60.8%) of those experiencing middle-of-night awakenings reporting poor sleep quality.

Notably, dream-related sleep disruptions showed a strong association with sleep quality (χ²=147.401, p<0.001), with 54 (79.4%) of participants experiencing bad dreams reporting poor sleep quality. The utilization of sleep medication demonstrated a particularly strong correlation with sleep quality (χ²=118.869, p<0.001), with 33 (97.1%) of those using self-medication reporting poor sleep quality.

Enthusiasm levels exhibited a significant relationship with sleep quality (χ²=143.440, p<0.001), where 494 (84.7%) of participants reporting enthusiasm experienced good sleep quality, contrasting sharply with only 14 (20.9%) among those lacking enthusiasm.

Interestingly, certain demographic variables showed no significant association with sleep quality, including gender (χ²=0.388, p=0.533), education level (χ²=0.099, p=0.952), and regional residence (χ²=0.527, p=0.468), suggesting these factors may not be crucial determinants of sleep quality in this population.

The logistic regression analysis in Table 4 revealed significant associations between poor sleep quality and multiple predictors. Self-medication for sleep emerged as the strongest predictor, with both crude (OR=11.23, 95% CI: 4.52-27.89, p<0.001) and AOR (AOR=8.45, 95% CI: 3.12-22.86, p<0.001) indicating robust association. Depression severity demonstrated a clear dose-response relationship, with moderately severe/severe depression showing notably elevated risk (AOR=7.92, 95% CI: 3.45-18.19, p<0.001) compared to minimal depression. Sleep initiation difficulty (AOR=3.75, 95% CI: 2.15-6.54, p<0.001) and lack of enthusiasm (AOR=4.15, 95% CI: 2.18-7.89, p<0.001) maintained significant associations after adjustment for confounders. The model demonstrated a good fit (Hosmer-Lemeshow χ²=9.12, p=0.38) with substantial explanatory power (Nagelkerke R²=0.56).

**Table 4:** Factors associated with poor sleep quality - multivariate logistic regression analysis Crude and AOR with 95% CI are presented. *Statistically significant at a p-value less than 0.05. AOR, adjusted odds ratio

Variables	Crude OR (95% CI)	p-value	AOR (95% CI)	p-value
Age (per year increase)	0.98 (0.96-0.99)	0.002*	0.97 (0.95-0.99)	0.003*
Unemployment (vs. employed)	2.78 (1.82-4.24)	<0.001*	2.31 (1.45-3.68)	<0.001*
Depression severity				
Minimal (reference)	1.00 (reference)	-	1.00 (reference)	-
Mild	3.45 (2.12-5.62)	<0.001*	2.84 (1.67-4.82)	<0.001*
Moderate	5.88 (3.21-10.77)	<0.001*	4.56 (2.35-8.86)	<0.001*
Moderately severe/ severe	9.64 (4.45-20.89)	<0.001*	7.92 (3.45-18.19)	<0.001*
Sleep initiation difficulty	5.12 (3.15-8.33)	<0.001*	3.75 (2.15-6.54)	<0.001*
Midnight awakening	3.95 (2.42-6.44)	<0.001*	2.89 (1.66-5.03)	<0.001*
Bad dreams	4.88 (2.75-8.66)	<0.001*	3.42 (1.78-6.57)	<0.001*
Self-medication for sleep	11.23 (4.52-27.89)	<0.001*	8.45 (3.12-22.86)	<0.001*
Lack of enthusiasm	5.67 (3.24-9.92)	<0.001*	4.15 (2.18-7.89)	<0.001*

Analysis across depression severity levels revealed progressive deterioration in sleep parameters as shown in Table 5. Mean PSQI scores increased systematically from minimal (3.2±1.8) to severe depression (11.3±3.2). Sleep latency exceeding 30 minutes was observed in 8.3% of participants with minimal depression, increasing to 56.2% in severe cases (p<0.001). Similar trends were noted for sleep duration <6 hours (12.5% to 59.4%, p<0.001) and sleep efficiency <85% (15.6% to 62.5%, p<0.001). Daytime dysfunction showed the most marked progression, from 18.2% in minimal depression to 78.1% in severe cases (p<0.001).

**Table 5:** Sleep quality parameters across depression severity levels *Statistically significant when the p-value was less than 0.05. PSQI, Pittsburgh Sleep Quality Index

Parameters	Minimal	Mild	Moderate	Severe	p-value
Mean PSQI score (SD)	3.2 (1.8)	5.7 (2.4)	8.1 (2.9)	11.3 (3.2)	<0.001*
Sleep latency >30 min (%)	8.3	18.7	35.5	56.2	<0.001*
Sleep duration <6 h (%)	12.5	23.4	41.9	59.4	<0.001*
Sleep efficiency <85% (%)	15.6	28.1	45.2	62.5	<0.001*
Daytime dysfunction (%)	18.2	35.7	58.1	78.1	<0.001*

The correlation matrix demonstrated significant relationships among key study variables as shown in Table 6. PSQI scores showed a strong positive correlation with PHQ-9 scores (r=0.65, p<0.001) and a moderate negative correlation with age (r=-0.22, p<0.001). Sleep latency exhibited a substantial correlation with PSQI scores (r=0.58, p<0.001) and a moderate correlation with PHQ-9 scores (r=0.45, p<0.001). Sleep efficiency and duration showed a moderate positive correlation (r=0.56, p<0.001), while both parameters demonstrated inverse relationships with sleep latency (r=-0.38 and r=-0.42 respectively, p<0.001 for both).

**Table 6:** Correlation matrix of key study variables PSQI, Pittsburgh Sleep Quality Index; PHQ-9, Patient Health Questionnaire-9

Variables	1	2	3	4	5	6
PSQI score	1	-	-	-	-	-
PHQ-9 score	0.65	1	-	-	-	-
Age	-0.22	-0.28	1	-	-	-
Sleep latency	0.58	0.45	-0.18	1	-	-
Sleep duration	-0.52	-0.38	0.15	-0.42	1	-
Sleep efficiency	-0.48	-0.35	0.12	-0.38	0.56	1

## Discussion

This cross-sectional study provides substantial evidence regarding the complex interrelationship between depression and sleep quality among the general population of Perambalur, Tamil Nadu. The study revealed that 21.8% of participants experienced poor sleep quality, while 40.8% exhibited varying degrees of depression, ranging from mild to severe. These findings align with recent research by Alfian et al. (2024), who reported similar prevalence rates in the Indonesian general population, though their study showed slightly higher rates of sleep disturbances (25.3%) [9]. Similar patterns were observed by Drager et al. (2022) in their cross-sectional study of the Brazilian general population, although they reported a higher prevalence of poor sleep quality (32.9%), possibly due to different socio-cultural contexts [8].

Our observed 21.8% prevalence of poor sleep quality and 40.8% depression rate can be meaningfully contextualized within the Indian epidemiological landscape through Mittal et al.'s (2024) cross-sectional investigation of cognitive functioning in adults with chronic insomnia disorder, which established similar psychopathological patterns in an Indian clinical population [18]. The progressive deterioration in sleep parameters across depression severity levels observed in our study aligns with Brick et al.'s (2010) findings on the association between sleep hygiene and sleep quality in medical students [17].

The significant association between depression severity and sleep quality parameters demonstrates a clear dose-response relationship, with more severe depression correlating with poorer sleep outcomes. This relationship is particularly evident in the progressive deterioration of sleep parameters across depression severity levels, where PSQI scores increased systematically from minimal (3.2±1.8) to severe depression (11.3±3.2). These findings correspond with the work of Menoscal et al. (2024) in the ELSI-Brazil cohort, though their study focused specifically on older adults [19]. The association is further supported by Biswas et al. (2024), who found similar patterns among geriatric populations in West Bengal, particularly regarding sleep initiation difficulties and maintenance [20].

The strong positive correlation between PSQI and PHQ-9 scores (r=0.65, p<0.001) supports the bidirectional relationship between sleep disturbances and depression, as previously documented by Mazza et al. (2005) and recently reinforced by Cho and Kim (2023) in their study of T1DM patients [21,22]. This finding is particularly noteworthy in light of Du et al.'s (2024) recent NHANES study, which emphasized the interconnected nature of sleep duration, dietary quality, and depression symptoms in the general population [23].

The impact of sleep quality on daily functioning revealed in our study aligns with the findings of Killgore (2010) and Tucker et al. (2010), who demonstrated the significant effects of sleep deprivation on cognitive function and executive functioning [3,4]. The progressive increase in daytime dysfunction from 18.2% in minimal depression to 78.1% in severe cases particularly resonates with Harrison et al.'s (2000) research on sleep deprivation's impact on decision-making capabilities [5]. These findings are further supported by Carpi and Vestri (2022), who demonstrated the mediating role of sleep quality in the relationship between negative emotional states and health-related quality of life [13].

Our findings regarding sociodemographic factors reveal interesting patterns that both support and challenge existing literature. The significant age-related differences in depression and sleep quality, with younger participants showing higher vulnerability to both conditions, align with the findings of Sahaya Sowmiya et al. (2024) and Alotaibi et al. (2020), who reported similar age-related patterns among healthcare professionals [10,12]. This age-related pattern is particularly interesting when considered alongside the findings of Cates et al. (2015) and Giri et al. (2013), who documented similar vulnerabilities among young adult populations, specifically medical and pharmacy students [11,24]. However, our observation of higher depression rates in urban residents (45.1%) compared to rural residents (34.6%) presents a contrast to some previous studies, possibly reflecting unique regional characteristics or urbanization-related stressors in the study area.

The occupational status findings, showing higher rates of poor sleep quality and depression among unemployed individuals, correspond with broader literature on the psychological impact of unemployment. This association might be particularly relevant in the context of developing economies, as suggested by Mohammed Salih et al. (2023) in their cross-sectional study [25]. The relationship between occupational stress and sleep quality has been further elaborated by Morin et al. (2003), who emphasized the role of stress and arousal in primary insomnia [6]. These findings gain additional context when considered alongside Zunhammer et al.'s (2014) research on sleep quality during periods of stress, which highlighted the complex interplay between psychological stress and sleep disturbances [26]. Additionally, our occupational status findings correspond with existing Indian research on the psychological impact of employment status, as documented by Giri et al. (2013) in their study of sleep habits among medical students in Western Maharashtra [24]. The strong correlation between enthusiasm levels and sleep quality (χ²=143.440, p<0.001) gains particular relevance when considered alongside Sahaya Sowmiya et al.'s (2024) research on the impact of depression, anxiety, and occupational stress on sleep quality among dental practitioners in Madurai [12], providing a regionally specific comparative framework that strengthens the ecological validity of our findings within the South Indian context.

The analysis of sleep-related behavioral parameters revealed strong associations with both depression and overall sleep quality, particularly regarding sleep initiation difficulties, nocturnal awakenings, and use of sleep medication. These findings support the work of Steiger and Pawlowski (2019) and are consistent with Pigeon et al.'s (2008) research on the perpetuating factors of late-life depression [2,7]. The high prevalence of self-medication for sleep problems in our study (5.2%) aligns with concerns raised by Almojali et al. (2017) and Alsaggaf et al. (2016) regarding stress-related sleep disturbances and their management among various population groups [27,28].

The impact of poor sleep quality on overall health outcomes suggested by our findings resonates with recent research by Antza et al. (2021), who documented links between sleep duration and metabolic disorders, and Palagini et al. (2013), who established connections between sleep loss and hypertension [14,15]. These associations underscore the broader public health implications of our findings, particularly given the high prevalence of both conditions in our study population. The findings are further contextualized by Camilo's (2024) recent literature review on the impact of sleep deprivation in depression treatment, suggesting potential therapeutic implications for addressing both conditions simultaneously [29].

Clinical significance

The clinical significance of this study lies in its comprehensive examination of the depression-sleep quality relationship within a general population context, offering valuable insights for healthcare providers and public health practitioners. The identified dose-response relationship between depression severity and sleep quality parameters provides a crucial framework for clinical assessment and intervention strategies.

The finding that self-medication for sleep emerged as the strongest predictor of poor sleep quality (AOR=8.45, 95% CI: 3.12-22.86) highlights an urgent need for healthcare providers to address sleep problems through evidence-based interventions rather than allowing patients to resort to self-medication. This is particularly relevant given the potential risks associated with unmonitored use of sleep aids.

The strong association between lack of enthusiasm and both depression and poor sleep quality suggests the importance of screening for these conditions in patients presenting with reduced motivation or energy levels. The progressive deterioration in sleep parameters across depression severity levels provides clinicians with objective markers for monitoring disease progression and treatment response.

Furthermore, the identified sociodemographic risk factors, particularly the higher vulnerability of younger urban residents and unemployed individuals, can help healthcare providers identify at-risk populations for targeted screening and intervention. The findings also emphasize the need for integrated treatment approaches that address both sleep disturbances and depressive symptoms simultaneously.

Strength of the study

The study's primary strengths lie in its methodologically robust approach and comprehensive assessment of multiple variables. The use of validated assessment tools (PSQI and PHQ-9) ensures the reliability of the findings. The large sample size (n=650) and diverse sociodemographic representation enhance the generalizability of the results within the study region.

The inclusion of both urban and rural populations provides valuable comparative data, while the detailed analysis of sleep-related parameters offers insights into specific aspects of sleep disturbance. The strong statistical approach, including multivariate analysis and control for confounding factors, strengthens the validity of the findings. The high response rate and face-to-face interview methodology helped minimize response bias and ensure data quality.

Limitations

Despite its strengths, several limitations warrant consideration. The cross-sectional design prevents the establishment of causal relationships between depression and sleep quality. The self-reported nature of the data may introduce recall bias, particularly regarding sleep parameters. The study's single-region focus may limit generalizability to other geographical or cultural contexts. The absence of objective sleep measurements (such as polysomnography) means that sleep quality assessment relies solely on subjective reports. Additional limitations include the reliance on convenience sampling, which may introduce selection bias despite stratification efforts. The study's cross-sectional timeframe (December 2022-February 2023) does not account for potential seasonal variations in sleep patterns and depression symptoms. Furthermore, the absence of data on comorbid physical conditions limits our understanding of potential confounding medical factors affecting both sleep quality and depression.

Recommendations

Based on our findings, we propose implementing structured depression and sleep quality screening protocols in primary healthcare settings, with particular emphasis on high-risk populations including younger urban residents and unemployed individuals. We recommend the development of interdisciplinary treatment frameworks that simultaneously address both depression and sleep disturbances, utilizing standardized screening tools (PHQ-9 and PSQI) for early detection and intervention. Healthcare systems should establish referral pathways integrating psychiatric and sleep medicine services to facilitate comprehensive patient management.

Future research initiatives should employ longitudinal methodologies to elucidate temporal relationships and causal mechanisms between depression and sleep parameters. Implementation of public health education programs focusing on sleep hygiene practices and depression awareness is warranted, particularly in urban settings. Community-based mental health interventions targeting unemployed populations should be prioritized, with concurrent development of evidence-based guidelines addressing self-medication for sleep disturbances. Healthcare policies should emphasize improved accessibility to specialized mental health services and sleep disorder clinics, incorporating standardized treatment protocols aligned with the bidirectional relationship between depression and sleep disturbances demonstrated in our findings.

## Conclusions

This comprehensive study establishes a strong association between depression and sleep quality in the general population of Perambalur, with clear evidence of a dose-response relationship between depression severity and sleep disturbances. The findings highlight the complex interplay of sociodemographic factors, particularly age, urbanization, and employment status, in influencing both conditions. The study's results emphasize the need for integrated approaches to mental health and sleep disorders in primary healthcare settings, while also identifying specific risk factors and vulnerable populations requiring targeted interventions. The high prevalence of self-medication for sleep problems underscores the importance of improving access to professional healthcare services. These findings contribute significantly to the understanding of depression-sleep quality relationships in general populations and provide valuable guidance for healthcare providers, public health practitioners, and policymakers in developing effective intervention strategies.
